# Sulfate-Induced Stomata Closure Requires the Canonical ABA Signal Transduction Machinery

**DOI:** 10.3390/plants8010021

**Published:** 2019-01-16

**Authors:** Hala Rajab, Muhammad Sayyar Khan, Mario Malagoli, Rüdiger Hell, Markus Wirtz

**Affiliations:** 1 Centre for Organismal Studies (COS), Heidelberg University, 69120 Heidelberg, Germany; hala.rajab@cos.uni-heidelberg.de (H.R.); ruediger.hell@cos.uni-heidelberg.de (R.H.); 2 Institute of Biotechnology and Genetic Engineering, The University of Agriculture Peshawar, 25000 Peshawar, Pakistan; sayyar@aup.edu.pk; 3 Department of Agronomy, Food, Natural Resources, Animals and Environment, University of Padova, 35020 Legnaro, Italy; mario.malagoli@unipd.it

**Keywords:** sulfate, abscisic acid, stomatal closure, phytohormone synthesis, NADPH oxidase, Protein phosphatases 2C, Sucrose non-fermenting Related Kinase 2 (SnRK2), reactive oxygen species (ROS)

## Abstract

Phytohormone abscisic acid (ABA) is the canonical trigger for stomatal closure upon abiotic stresses like drought. Soil-drying is known to facilitate root-to-shoot transport of sulfate. Remarkably, sulfate and sulfide—a downstream product of sulfate assimilation—have been independently shown to promote stomatal closure. For induction of stomatal closure, sulfate must be incorporated into cysteine, which triggers ABA biosynthesis by transcriptional activation of NCED3. Here, we apply reverse genetics to unravel if the canonical ABA signal transduction machinery is required for sulfate-induced stomata closure, and if cysteine biosynthesis is also mandatory for the induction of stomatal closure by the gasotransmitter sulfide. We provide genetic evidence for the importance of reactive oxygen species (ROS) production by the plasma membrane-localized NADPH oxidases, RBOHD, and RBOHF, during the sulfate-induced stomatal closure. In agreement with the established role of ROS as the second messenger of ABA-signaling, the SnRK2-type kinase OST1 and the protein phosphatase ABI1 are essential for sulfate-induced stomata closure. Finally, we show that sulfide fails to close stomata in a cysteine-biosynthesis depleted mutant. Our data support the hypothesis that the two mobile signals, sulfate and sulfide, induce stomatal closure by stimulating cysteine synthesis to trigger ABA production.

## 1. Introduction

Plants have to respond to diverse environmental challenges to optimize growth and ensure survival during stress. Regulation of the stomatal aperture is a critically controlled stress response of plants. Stomata are the gates of the plants for interaction with their environment, and various input signals such as pathogen attack, CO_2_-concentration, light, heat, humidity, and soil water supply, need to be integrated for the optimal opening of these pores. Phytohormone abscisic acid (ABA) is a potent regulator of stomatal aperture and has been shown to transduce many abiotic and biotic input signals for stomatal closure. 

The ABA signal transduction cascade is one of the best-characterized input transmission pathways of plants at the molecular level. In guard cells, ABA physically interacts with the PYRABACTIN RESISTANCE1 (PYR1) and PYR1-LIKE (PYL)-proteins or regulatory components of the ABA receptor (RCAR). Binding of ABA to PYR/PYL receptor enhances the affinity of PYR/PYL receptors for ABI1, a PROTEIN PHOSPHATASE of type 2C (PP2C). PP2Cs are inhibited after binding to the ABA-PYR/PYL receptor complex. Inactivation of PP2Cs by ABA causes activation of subclass III Sucrose non-fermenting Related Kinase 2 (SnRK2s) [1], of which SnRK2.6 (OPEN STOMATA 1, OST1, Q940H6) is most relevant for stomatal closure. OST1 is a very potent actor since it phosphorylates several targets whose activities contribute to stomatal closure. One of these targets is the SLOW ANION CHANNEL 1 (SLAC1, [2]). SLAC1-induced current changes result in activation of outward K+ channels. The K+ efflux decreases the osmotic potential in the guard cells, followed by water export. The resulting decreased turgor of the guard cell is the physical cause for stomatal is is closure [3]. Furthermore, OST1 can phosphorylate the plasma membrane-resident β-nicotinamide adenine dinucleotide 2′-phosphate (NADPH) oxidase RESPIRATORY BURST OXIDASE HOMOLOG F (RBOHF, O48538) at Ser^13^ and Ser^174^, which is crucial for the regulation of RBOHF activity [4]. NADPH oxidases produce apoplastic reactive oxygen species (ROS) that are essential for ABA-induced stomatal closure. Internal and apoplastic ROS affect multiple steps during ABA signaling and act as a second messenger that plays a predominant role as an ABA signal amplifier [5]. 

Several studies connect the drought stress response to the assimilation of sulfur [6,7]. Drought stress regulates the sulfur assimilation pathway in an organ-specific manner and causes differential accumulation of sulfur-metabolism related compounds of the primary sulfur metabolism (e.g., glutathione) and the secondary sulfur metabolism (e.g., 3′-phosphoadenosine 5′-phosphate, PAP) [7,8]. The ROS scavenger glutathione acts in the cytosol, the plastids, and the mitochondria as a redox buffer during stress-induced accumulation of ROS. Its drought-stress induced accumulation has been interpreted as a protection mechanism to avoid over-oxidation of these compartments upon stress-induced ROS formation [9,10,11,12]. 

In contrast, PAP acts as a redox stress-induced retrograde signal of the chloroplast in drought and high light signaling by affecting the expression of nuclear-encoded stress-related genes [8,13]. Recently, PAP has also been shown to act as a second messenger of ABA signaling during stomatal closure that bypasses the canonical ABA signaling components ABI1 and OST1 [8]. PAP is a byproduct of sulfation reactions catalyzed by cytosolic sulfotransferases that transfer the activated sulfate of 3′-phosphoadenosine 5′-phosphosulfate (PAPS) to various compounds [14]. The cytosolic PAP is counter exchanged with the predominantly plastid-generated PAPS and degraded in the plastids by the highly redox-regulated 3′(2′),5′-bisphosphate nucleotidase (EC 3.1.3.7, SAL1) [13,15]. 

Remarkably, sulfate is an early xylem-borne chemical signal in maize under soil drying conditions and precedes the root-to-shoot transport of ABA and the pH increase of the xylem sap [16]. ABA transport and an increase of xylem sap pH have long been assumed to serve as a root-to-shoot signal during soil drying. In Poplar, drought-stress also increases the concentration of sulfate in the xylem by lowered sulfate xylem unloading via PtaSULFATE TRANSPORTER 3;3a (PtaSULTR3;3a) and PtaSULTR1;1, and by enhanced sulfate loading from parenchyma cells into the xylem via ALUMINIUM ACTIVATED MALATE TRANSPORTER3b (PtaALMT3b). Furthermore, sulfate has been shown to decrease relative transpiration and stomatal conductance after petiole feeding of sulfate to detached Poplar leaves [17]. The studies established soil-drying induced xylem transport of sulfate as a candidate for the root-to-shoot signal of the water status but did not uncover the signal transduction pathway for sulfate-induced stomatal closure at the molecular level (see below). 

After xylem transport of sulfate to the leaves, the sulfate can be stored in the vacuole of leaf cells or transported into the plastids where it can be activated to APS by ATP sulfurylase [18]. APS is either substrate for production of PAPS by APS kinase or reduced to sulfide by subsequent action of APS REDUCTASE (APR) and SULFiTE REDUCTASE (SiR). The competition between APS reductase and APS kinase for their common substrate APS controls sulfur partitioning between the primary and secondary sulfur metabolism [19]. APR and SiR are exclusively localized in plastids and control the flux through the assimilatory sulfate reduction pathway which generates sulfide [20,21]. Three *O*-ACETYLSERINE-THIOL-LYASE (OAS-TL) isoforms catalyze in the plastids, the mitochondria, and the cytosol the incorporation of sulfide into cysteine, which is the precursor for all reduced sulfur-containing compounds in plants, e.g., glutathione [20,21,22,23,24]. The carbon backbone for incorporation of sulfide into cysteine is *O*-acetylserine (OAS), and is separately produced in all subcellular compartments with its own cysteine synthesis by five SERINE ACETYLTRANSFERASEs (SERATs, [25]). The subcellular localization of OAS-TL and SERAT implies that significant amounts of the membrane-permeable sulfide move from the plastids to the cytosol and the mitochondria for incorporation into cysteine [26,27].

Sulfide is highly toxic and efficiently incorporated into cysteine in mitochondria, which significantly contributes to the detoxification of elevated sulfide levels [28]. On the other hand, sulfide acts in humans and plants as a volatile gasotransmitter that controls various physiological responses [29,30]. In plants, sulfide represses autophagy and induces stomatal closure [31,32]. However, the mode of sulfide-induced stomatal closure is still controversially discussed. Sulfide has been suggested to (1) affect ABA receptor expression directly [33], (2) act upstream of nitric oxide (NO) to modulate ABA-dependent stomatal closure [34], (3) induce in a NO-dependent manner accumulation of 8-mercapto-cGMP for stomatal closure [35], or (4) activate SLAC1 in an OST1- dependent manner [31]. 

We recently showed that sulfate must be incorporated into cysteine to trigger stomata dynamics. Consequently, sulfate-induced stomata closure was impaired in mutants deficient in the synthesis of cysteine or ABA [36]. Remarkably, cysteine synthesis depleted mutants are sensitive to drought and high light stress [36,37,38]. Both stresses also cause PAP accumulation. Since sulfide is a downstream product of assimilatory sulfate reduction pathway and PAP formation is a result of sulfation reactions, it is important to dissect how PAP, sulfide, and sulfate control stomatal aperture.

Here, we apply reverse genetics to understand the contribution of the canonical ABA signaling machinery to sulfate-induced stomata closure and dissect the potential relevance of the sulfation byproduct PAP in this process. We found that the protein phosphatase ABI1 and the down-stream kinase OST1 are essential for sulfate-induced ROS formation in stomata and stomatal closure. Since PAP acts independently of OST1, we concluded that potential accumulation of PAP upon external sulfate administration does not significantly contribute to sulfate-induced stomatal closure. In concordance with the function of ROS as an amplifier of ABA signaling, the loss-of-function mutants for the NADPH oxidases RBOHD and RBOHF are also impaired in sulfate-induced stomatal closure. We furthermore demonstrate that sulfide-induced stomatal closure requires the presence of the major SERAT isoforms located in the cytosol, the plastids, and the mitochondria, strongly suggesting that sulfide needs to be integrated into cysteine to promote stomatal closure. We suggest that sulfate and sulfide are incorporated into cysteine to trigger ABA formation, which in turn requires canonical ABA signaling components to mediate sulfate/sulfide/cysteine-induced stomatal closure.

## 2. Results 

In our previous study, we demonstrated that sulfate can close stomata and that it needs to be incorporated into cysteine for activation of ABA synthesis and accumulation of ABA in the cytosol of guard cells. However, we did not show how sulfate-induced ABA is perceived to trigger stomatal closure. 

### 2.1. Sulfate-induced Stomatal Closure Requires Functional ABA Signaling 

**Figure 1:**
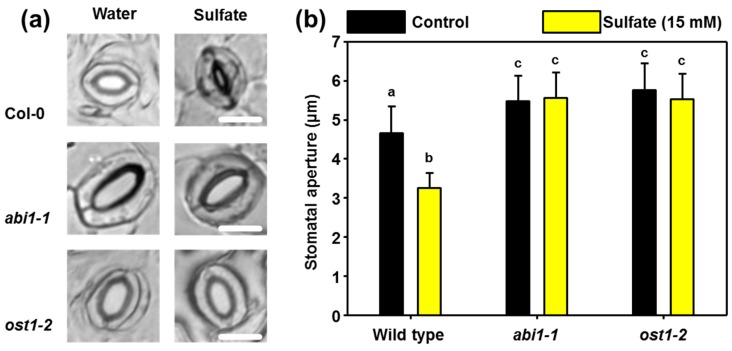
Functional ABI1 and OST1 are essential for sulfate-induced stomatal closure in Arabidopsis. (**a**) Representative guard cell in epidermal peels of 5-weeks–old soil-grown wild-type, *abi1-1*, and *ost1-2* plants that were floated on water at pH 5.5 or water supplemented with sulfate (15 mM MgSO_4_) for three hours. (**b**) Quantification of the stomatal aperture of guard cells in epidermal peels from plants indicated in (**a**) that were treated with water (control, black) supplemented with sulfate (15 mM, yellow). Letters indicate statistically significant differences between groups determined with the one Way ANOVA test (*p* < 0.05, n = 50 stomata, derived from epidermal peels of five individual plants). Values represent means ± standard deviation (SD). Scale bar, 10 µm.

In order to provide direct evidence for the biological relevance of the ABI1 during sulfate-induced stomata closure, we challenged epidermal peels of 5-week-old soil grown wild-type and *abi1-1* plants with 15 mM sulfate for three hours. In the *abi1-1* mutant, the protein phosphatase ABI1 is constitutively active, which results in a permanent open stomata phenotype ([39,40], Figure 1a). Application of 15 mM sulfate efficiently closed the stomata of wild-type plants (Figure 1a), which supports the previously reported impact of sulfate on stomatal closure [17,36]. In contrast, stomata did not close in the *abi1-1* mutant after application of sulfate, demonstrating that sulfate-induced ABA accumulation in guard cells is not triggering stomatal closure when the PYR/PYL-ABA-ABI1 ternary complex is non-functional (Figure 1a,b). 

**Figure 2:**
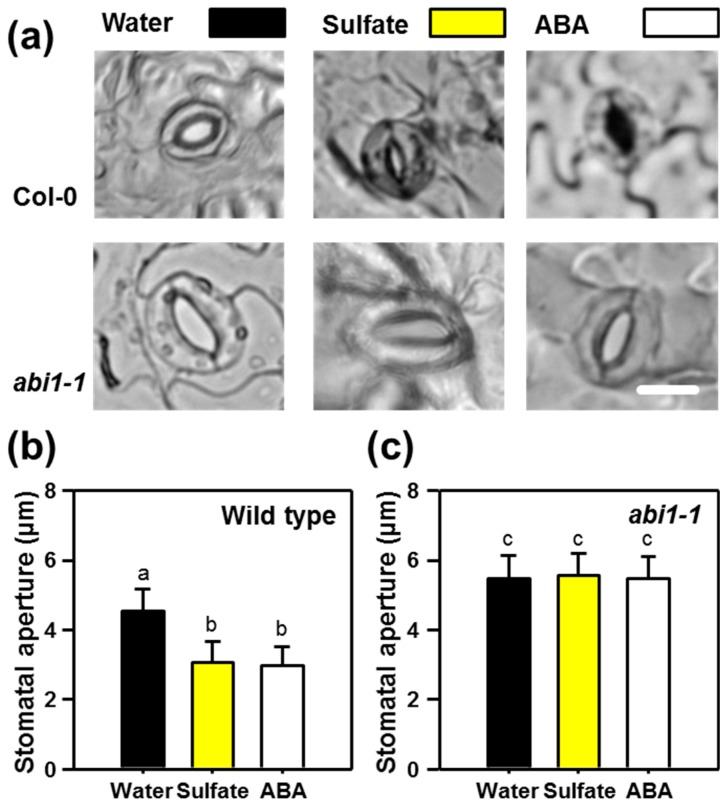
Functional ABI1 is required for stomatal closure upon ABA or sulfate treatment. (**a**) Representative guard cell in epidermal peels of 5-week–old soil-grown wild-type and *abi1-1* plants that were floated on water at pH 5.5 or water supplemented with sulfate (15 mM MgSO_4_) or ABA (50 µM) for three hours. (**b**,**c**) Quantification of the stomatal aperture in epidermal peels of the wild-type (**b**) and the *abi1-1* mutant (**c**) that were treated with water (control, black) supplemented with sulfate (15 mM, yellow) or ABA (50 µM, white). Letters indicate statistically significant differences between groups determined with the one Way ANOVA test (*p* < 0.05, n = 50 stomata, derived from epidermal peels of five individual plants). Values represent means ± standard deviation (SD). Scale bar, 10 µm.

In a separate experiment, we independently confirmed the absence of sulfate responsiveness for *abi1-1* and applied ABA as a control for stomatal closure. Treatment of sulfate (15 mM) and ABA (50 µM) resulted in significant stomatal closure in the wild-type. The degree of stomatal closure was indistinguishable after sulfate and ABA application. ABA or sulfate application to epidermal peels of the *abi1-1* mutant did not affect the stomatal aperture (Figure 2).

Binding of ABA to PYR/PYL receptors causes efficient inactivation of the PP2C named ABA INSENSITIVE1 (ABI1). The inactivation of ABI1 releases the downstream kinase OST1 from inhibition, which in turn phosphorylates multiple targets to promote ABA-induced stomatal closure. In the *ost1-2* mutant, a T-DNA insertion in the OST1 gene destabilizes the OST1 mRNA, resulting in a total loss-of-OST1 function. Like the *abi1-1* mutant, *ost1-2* displays constitutively open stomata and is sensitive to soil drying and low humidity ([41], Figure 1a). The absence of OST1 kinase activity inhibits the impact of sulfate on stomatal aperture (Figure 1a,b), which is in concordance with the previously demonstrated function of sulfate for the promotion of ABA accumulation in guard cells [36]. These results suggest that the PYR/PYL receptors sense sulfate-induced accumulation of ABA in guard cells and support the importance of the ABI1-OST1 phosphorylation relay for sulfate-induced stomatal closure. 

### 2.2. ABI1 and OST1 are Essential for the Sulfate-induced Formation of ROS in Guard Cells 

**Figure 3:**
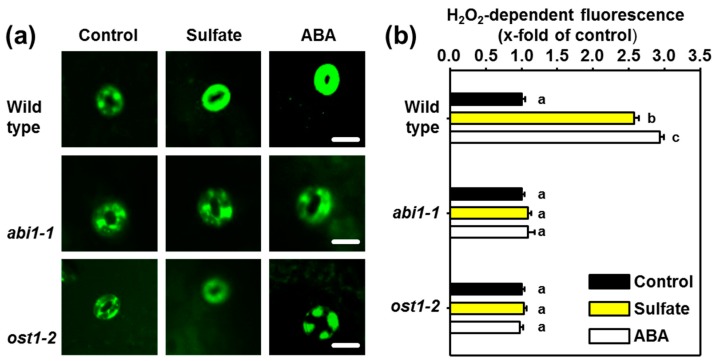
Functional ABI1 and OST1 are vital for the sulfate-induced accumulation of reactive oxygen species in guard cells. (**a**) Visualization of reactive oxygen species (ROS) with the H_2_O_2_-selective dye 2′,7′-dichlorofluorescein (H2DCF) as described in material and methods. Epidermal peels of 5-weeks–old soil-grown wild-type, *abi1-1*, and *ost1-2* plants were floated on water at pH 5.5 or water supplemented with sulfate (15 mM MgSO_4_) or ABA (50 µM) for three hours prior staining of ROS. (**b**) Quantification of ROS staining in guard cells floated on water (control, black) supplemented with sulfate (15 mM, yellow) or ABA (50 µM, white). Letters indicate statistically significant differences between groups determined with the one way ANOVA test (*p* < 0.05, n = 50 stomata, derived from epidermal peels of five individual plants). Values represent means ± standard deviation (SD). Scale bar, 10 µm.

A known target of the ABI1-OST1 phosphorylation relay is the membrane resident NADPH oxidase RBOHF, which is essential for ABA-induced ROS production [4]. We, therefore, tested the formation of ROS in guard cells after sulfate-application to epidermal peels from the wild-type, the *abi1-1*, and the *ost1-2* mutant. Application of sulfate (15 mM) resulted in significant production of ROS in the wild type. The degree of ROS formation in response to sulfate was comparable to the formation of ROS after application of 50 µM ABA (Figure 3a,b). The guard cells in epidermal peels of *abi1-1* and *ost1-2* displayed wild-type like levels of ROS under control conditions. Both mutants of the ABI1-OST1 phosphorylation relay failed to produce ROS in response to the application of sulfate, which is in concordance with the failure to close stomata in response to sulfate. Also, ABA application did not induce ROS formation in both mutants under their applied conditions, which is in agreement with results of previous studies [5].

### 2.3. Sulfate Stimulus Activates NADPH Oxidases for Production of ROS in Guard Cells

The sulfate-induced accumulation of ROS in wild-type guard cells has also been observed in our previous study on sulfate-induced stomatal closure [36]. In this study, we demonstrated that inhibition of oxidases, like RBOH isoforms A-F, with the selective inhibitor diphenyleneiodonium prevents the sulfate-induced formation of ROS in guard cells. Co-application of sulfate and diphenyleneiodonium also impaired sulfate-induced stomatal closure, strongly suggesting that formation of ROS is vital for transduction of the sulfate stimulus. These findings support the hypothesis that sulfate induces synthesis of ABA in guard cells, which causes the formation of the ABA-PYR/PYL-ABI1 ternary complex resulting in activation of OST1. The activated OST1 potentially phosphorylates NADPH oxidases to produce the second messenger ROS. The elevated ROS levels will act as a signal amplifier to activate SLAC1, leading to stomatal closure upon sulfate stimulus [5].

**Figure 4:**
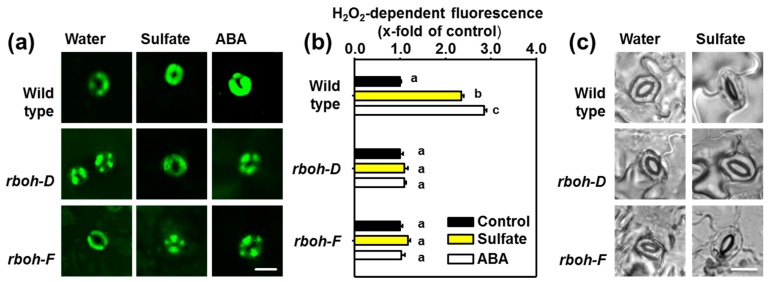
Sulfate-induced stomatal closure and ROS formation require the NADPH oxidases RBOHD, and RBOHF (**a**) Visualization of reactive oxygen species (ROS) with the H_2_O_2_-selective dye 2′,7′-dichlorofluorescein (H2DCF) as described in material and methods. Epidermal peels of 5-weeks–old soil-grown wild-type, *rboh-D*, and *rboh-F* plants were floated on water at pH 5.5 or water supplemented with sulfate (15 mM MgSO_4_) or ABA (50 µM) for three hours prior staining of ROS. (**b**) Quantification of ROS staining in guard cells floated on water (control, black) supplemented with sulfate (15 mM, yellow) or ABA (50 µM, white). Letters indicate statistically significant differences between groups determined with the one Way ANOVA test (*p* < 0.05, n = 50 stomata, derived from epidermal peels of five individual plants). Values represent means ± standard deviation (SD). (**c**) Representative guard cells embedded in epidermal peels of the wild-type and the *rboh-D*, and *rboh-F* mutants after treatment with water or water supplemented with sulfate (15 mM). Scale bar, 10 µm.

In order to link the activation of OST1 and the formation of ROS upon sulfate stimulus with the membrane resident NADPH oxidases, we tested the contribution of two major isoforms of RBOHs expressed in guard cells, RBOH-D and RBOH-F, to sulfate-induced ROS formation [42]. The *rboh-D* and *rboh-F* mutant lack functional isoforms of the NADPH oxidase due to a T-DNA insertion in the respective gene. The absence of either RBOH-D or RBOH-F impaired ROS formation in guard cells upon application of ABA or sulfate for three hours to epidermal peels (Figure 4a,b). Consequently, sulfate did not affect stomatal aperture in both mutants (Figure 4c). Wild-type guard cells produced ROS and closed stomata upon sulfate application.

### 2.4. Stomata of the Serat tko Mutant do not Close upon Sulfide Application 

**Figure 5:**
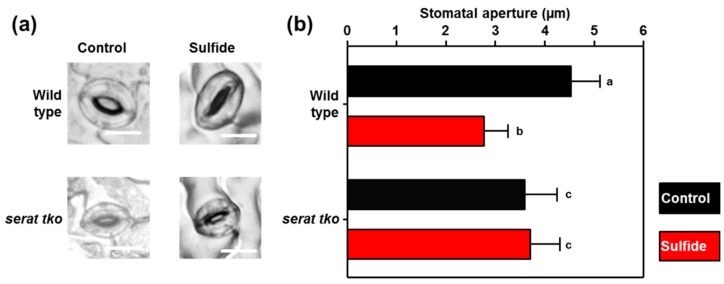
Sulfide is unable to induce stomatal closure in the OAS biosynthesis-depleted *serat tko* mutant. (**a**) Representative guard cells embedded in epidermal peels of the wild-type and the *serat tko* mutant after treatment with water at pH 5.5 (Control) or water supplemented with sulfide (100 µM NaHS) at pH 5.5 for 90 minutes. (**b**) Quantification of the stomatal aperture in epidermal peels of the wild-type and the *serat tko* mutant that were treated with water (control, black), or water supplemented with sulfide (100 µM NaHS, red). Letters indicate statistically significant differences between groups determined with the one Way ANOVA test (*p* < 0.05, n = 50 stomata, derived from epidermal peels of five individual plants). Values represent means ± standard deviation (SD). Scale bar, 10 µm.

The volatile signal H_2_S is a downstream product of sulfate assimilation and has been shown independently to induce stomatal closure [34,43,44]. Since sulfate must be incorporated into cysteine to gain competence as an inducer of stomatal closure, we decided to test if sulfide is also incorporated into cysteine for induction of ABA biosynthesis. Sulfide is the direct sulfur-precursor of cysteine and is incorporated by the activity of OAS-TL into cysteine. Cysteine biosynthesis is not limited by OAS-TL activity, but by the formation of the carbon skeleton for cysteine, OAS. The three major SERAT isoforms SERAT1;1, SERAT2;1 and SERAT2;2 produce the bulk of OAS for the incorporation of sulfide into cysteine in the cytosol, the plastids, and the mitochondria. A SERAT triple knock-out mutant (*serat tko*) lacking these major SERAT isoforms is retarded in growth and displays significantly lowered translation due to decreased production of OAS and cysteine [45]. When we applied 100 µM sulfide (NaHS) dissolved in water at pH 5.5 to epidermal peels of the wild-type, the dissolved sulfide triggered the closure of the wild-type stomata within 90 min. The application of water at pH 5.5 for the same duration did not affect the stomatal aperture (Figure 5). Remarkably, sulfide failed to close the stomata in epidermal peels of the *serat tko* mutant. These results suggest that sulfide is not perceived by a membrane-resident receptor located at the plasma membrane, but is incorporated into cysteine to gain competence as a stomatal closure signal.

## 3. Discussion

### 3.1. Sulfate and Sulfide are Incorporated into Cysteine to Trigger Stomatal Closure

Stomatal closure is a dynamic process that optimizes carbon dioxide uptake with transpiration-mediated water loss. A multifaceted signaling network regulates stomatal movements and integrates diverse environmental and endogenous inputs. Some of these inputs are locally generated, e.g., pathogen-induced stomatal closure, and cause fast responses that use phosphorylation relays to control plasma-membrane resident NADPH oxidases [5]. In other cases, e.g., perceiving of the soil-water status, a distantly originated signal must travel to the guard cell and will be integrated to modulate stomatal aperture. Many of these integration processes impinge on the ABA signal transduction pathway [3,46]. However, our knowledge of the regulation of the tissue-specific ABA biosynthesis sites in response to environmental cues is currently quickly evolving, but still limited [3,46]. 

Characterization of the plastidic sulfate transporter SULTR3;1 uncovered for the first time a direct link between the actual sulfur supply and ABA biosynthesis in plants [47,48]. Indeed, sulfate has been shown by independent groups to accumulate in the xylem sap of drought-stress maize, Poplar, common hop and Arabidopsis plants and was supposed to act as an early signal that promotes ABA-induced stomatal closure [16,17,49,50]. The recent identification of cysteine as a trigger for ABA biosynthesis in guard cells linked the sulfate-induced stomatal closure with the biosynthesis of this factor for stomatal closure [36]. The presented findings establish ABI1 as a required transducer of the ABA signal during sulfate-induced stomatal closure (Figure 1), which is in agreement with the stimulation of ABA production by cysteine and sulfate in guard cells and rosette leaves [36].

After perception, the ABA signal is transduced via the ABI1-OST1 phosphorylation relay to multiple downstream effectors. In concordance with this canonical view on ABA perception, OST1 is also vital for sulfate-induced stomatal closure. Both results strongly support the view that sulfate stimulates ABA formation and point against a direct gating of the QUICK ANION CHANNEL 1 (QUAC1 or ALMT12) channel by sulfate, which has been hypothesized by Malcheska and co-workers as the molecular basis of sulfate-induced stomata closure. This hypothesis was based on the stimulating effect of sulfate on QUAC1 expressed in *Xenopus* oocytes and the absence of stomatal closure in the *quac1* mutant [17]. The presented findings are not in contradiction with the insensitivity of the *quac1* mutant towards sulfate as a trigger of stomatal closure [17], but offer a different interpretation of the *quac1* insensitivity towards sulfate. Like SLAC1, QUAC1 is required for ABA-induced stomatal closure and is a substrate of OST1, which can activate SLAC1 and QUAC1 by direct phosphorylation [2,51,52]. Consequently, sulfate fails to close stomata in *quac1* [17] and *slac1-1* [36], although SLAC1 is not gated by sulfate.

Sulfide is a well-established signal for stomatal closure. Like sulfate, sulfide can be incorporated into cysteine. The failure of sulfide to stimulate stomatal closure in the *serat tko* (Figure 5), which is impaired in the incorporation of sulfide into cysteine, suggests that sulfate and sulfide use the same mechanism for induction of stomatal closure. Both signals stimulate the synthesis of cysteine, which in turn promotes ABA formation. In support of this hypothesis, sulfide is known to be immediately incorporated into cysteine after short-term exposure [27], and stimulates SLAC1 by activation of OST1 [31]. Furthermore, sulfate fails to close stomata in the *sir1-1* mutant that is depleted in its capability to reduce sulfate to sulfide [36,53]. 

### 3.2. The Role of ROS as Second-messenger in Sulfate-induced Stomatal Closure

ROS are important signal amplifiers of the ABA signal and act downstream of ABA as second messengers during stomatal closure and systemic acquired acclimation [5,54]. Like sulfide [55], sulfate application also triggered ROS production in guard cells of the wild type (Figure 3) in an RBOH dependent manner (Figure 4). The formation of ROS is essential for sulfate-induced stomatal closure [36]. The ABA-triggered ROS production in guard cells is OST1 dependent [41], which can directly phosphorylate plasma membrane-resident NADPH oxidases, like RBOH-F [4]. In concordance with the established scheme for ABA-induced ROS formation, sulfate-induced ROS production was diminished in *abi1-1*, *ost1-2* and loss-of-function mutants for NADPH oxidases (RBOH-D and RBOH-F). Remarkably, ABA-triggered formation of ROS depends on ABI1 but not on ABI2 [56]. Indeed, *abi2-1* mutants accumulated ROS in response to sulfate application [36]. Thus, the here presented data strongly suggest that the sulfate-induced ROS formation is a consequence of sulfate-promoted ABA formation, which in turn stimulates membrane resident NADPH oxidases via the classical ABA signal transduction cascade.

### 3.3. Contribution of Cytosolic Sulfation Reactions Releasing PAP to Close Stomata in Response to Sulfate

PAP is a side-product of sulfation reactions that use PAPs as sulfate donors [14]. PAP is also a potent inducer of stomatal closure [57]. Consequently, one could have hypothesized that sulfate-triggered PAP accumulation might contribute to sulfate-induced stomatal closure. Remarkably, PAP bypasses the canonical ABA-induced stomatal closure pathway and is independent of ABI1 and OST1 [57]. In contrast, sulfate-induced stomatal closure requires functional ABI1 and OST1 (Figure 1, Figure 2 and Figure 3) and its downstream targets (RBOH-F, Figure 4). Our findings exclude a significant contribution of PAP during sulfate-induced stomatal closure. Accumulation of PAP upon sulfate application is also highly questionable, since PAP levels are strongly regulated by the PAP degrading enzyme, SAL1 [6,8]. SAL1 is highly regulated by environmental stimuli like drought and high-light stress and tightly controls PAP level, which is the basis for the well-established retrograde signaling function of PAP [8,13].

In conclusion, our results uncover that sulfate-induced formation of the stomatal closure signal, ABA, is transduced by ABI1, which has been previously shown to physically interact with the PYR/PYL receptor in an ABA-dependent manner. The canonical ABI1-OST1 phosphorylation relay is essential for the activation of plasma-membrane resident NADPH oxidases of the RBOH type. These RBOHs produce the ABA signal amplifier ROS, which is mandatory for sulfate-induced stomatal closure. The failure of sulfide to close stomata in the *serat tko* mutant supports the view that sulfate and sulfide must be incorporated into cysteine to gain competences as stomatal closure signals due to the stimulation of ABA production.

## 4. Materials and Methods 

### 4.1. Plant Material and Growth

Seeds of *Arabidopsis thaliana* Col-0 (ecotype Columbia) and the mutants *abi1-1* (AT4G26080) CS22, *ost1-2* (AT4G33950), *rboh-D* (AT5G47910) CS9555, *rboh-F* (AT1G64060) CS68522, *serat tko* (SALK_ 050213 x SALK_ 099019 x Kazusa_KG752, [45]), were sown on soil (Tonsubstrat from Ökohum, Herbertingen) supplemented with 10% (v/v) vermiculite and 2% quartz sand. Seeds were stratified at 4 °C for two days in the dark. The plants were grown under long-day conditions for five weeks before the experiment (16 h light, 100 µmol m^−2^ s^−1^ at 22 °C and eight h dark at 18 °C for day and night, respectively). Relative humidity was set at 50%.

### 4.2. Stomatal Aperture Bioassay

Epidermal peels were prepared from the abaxial side of Arabidopsis leaves as described in [16] and allowed to float on distilled water for 2 hours under constant light. The peels were then transferred to distilled water pH 5.5 supplemented without (control) or with effectors (15mM MgSO_4_ and 50 µM ABA) for the indicated periods. Images of the stomata were captured after the treatment with a conventional wide-angle microscope (Leica DMIRB). The stomatal aperture was determined with ImageJ (http://fiji.sc/), using a µm ruler for calibration.

### 4.3. H_2_O_2_ Quantification in Guard Cells

ROS were determined in intact stomata of epidermal peels from the abaxial side of the leaf according to [58]. The peels were allowed to float on water without and with effectors for up to three hours. Subsequently, the epidermal peels were stained with 50 µM 2′,7′-dichlorodihydrofluorescein diacetate (H_2_DCFA) for 10 min and transferred to a microscope slide. The ROS-specific fluorescence was detected at 525 nm after specific excitation at 488 nm using a confocal microscope (Nikon A1R) according to [59]. Images were captured from peels of five individual plants, and the fluorescent signal of intact stomata (50) was quantified using the open source software ImageJ (http://fiji.sc/).

### 4.4. Statistical Analysis

The Statistical analysis for the experimental data was done using SigmaPlot 12.5 (Systat Inc., San Jose, CA, USA). The analysis of all data sets was analyzed through One Way Repeated Measures Analysis of Variance (one-Way ANOVA) for all of the multiple pairwise comparisons using the Holm-Sidak method. The Shapiro-Wilk method (p to reject was *p* > 0.05) was used to test the normality distribution of data. In the figures, letters are used to indicate the significance difference (*p* < 0.05).
